# Factors Associated with Postpartum Sexual Function During the Puerperium Period: A Cross-Sectional Study in Greece

**DOI:** 10.3390/nursrep15030086

**Published:** 2025-03-03

**Authors:** Christiana Arampatzi, Vasiliki Michou, Panagiotis Eskitzis, Konstantinos Andreou, Loukas Athanasiadis

**Affiliations:** 1 Department of Obstetrics, University of Western Macedonia, 50100 Kozani, Greece; xristianaarampatzi@gmail.com (C.A.); peskitzis@uowm.gr (P.E.); kandreou1@yahoo.gr (K.A.); 2 First Department of Psychiatry, Medical School, Aristotle University of Thessaloniki, 54124 Thessaloniki, Greece; loukatha@auth.gr

**Keywords:** puerperium, sexual health, sexuality, partner support, breastfeeding

## Abstract

**Background:** After childbirth, sexual activity and sexual desire decrease significantly. In recent years, postpartum sexual health has been a common concern that is often not discussed in antenatal postpartum care and has received little attention from either clinicians or researchers. This lack of attention is concerning, and thus, the aim of this study was to investigate associated factors with postpartum sexual function during the puerperium period, with the hope of sparking a change in this trend. **Methods:** Three hundred and thirty-six women participated in the study. They were asked to complete two questionnaires: a general questionnaire regarding demographic and other personal information about the postpartum period and the Female Sexual Functioning Index (FSFI). **Results:** The results showed that women scored an average of 20.8 points on the FSFI, and thus, their level of sexual functioning was characterized as moderate. Factors such as older age, lack of partner support, and negative body image appeared to influence scores on the FSFI scale. **Conclusions:** In conclusion, our research underscores the need for further investigation into the challenges women face during the puerperium period, which may negatively influence sexual health and functioning.

## 1. Introduction

Sexual health, as defined by the World Health Organization (WHO), is a comprehensive state of well-being. It encompasses not only physical health but also emotional, mental, and social well-being. It is more than just the absence of disease, dysfunction, or disability. Maintaining sexual health requires a positive attitude towards sexuality and relationships. It involves having safe, enjoyable sexual experiences free from coercion and discrimination. Most importantly, everyone’s sexual rights must be recognized and protected [1]. Sexuality is fundamentally connected to sexual health. Recognized as a basic human need, sexuality serves as a core element of sexual health, encompassing three key functions: reproduction, pleasure, and communication [2].

Sexuality is a fascinating and complex evolving process, coordinated by the nervous, vascular, and endocrine systems and shaped throughout life. It includes not only gender but also sexual identity and orientation, eroticism, pleasure from contact, and intimacy. It is influenced by biological, psychological, social, and economic variables; family and religious beliefs, etc. Sexuality is embodied differently within cultures through conventions; assigned roles and behaviors that express sexual desires; and various relationships of emotion and power based on beliefs, values, attitudes, emotions, and position in society. It also declines with aging and is interrelated with the health status and situations experienced by the individual. That is why the incidence of sexual dysfunction increases as women age. Sexual health both influences and is influenced by other health conditions, such as depression. Interestingly, it has been reported that those with sexual dysfunction should be screened for depression, and those showing depressive symptoms should also be evaluated for sexual dysfunction [3]. Also, its rates increase in the postpartum period, as important life events and experiences such as pregnancy, childbirth, and motherhood influence sexuality [4,5].

Pregnancy and childbirth result in numerous changes affecting the health and quality of life of new mothers. Research shows that pregnancy and childbirth lead to biological, psychological, and social changes that can significantly impact a woman’s sexual health and functioning. Notably, evidence indicates that sexual function often declines during pregnancy and may not fully return to its previous levels after childbirth. In a prospective study conducted in Sweden from 2014 to 2017, it was found that the percentage of sexually active women significantly decreased after childbirth, dropping from 98% at the start of pregnancy to 66.7% following delivery. However, this percentage rebounded to 90.0% 12 months after childbirth [6]. The prevalence of sexual disorders in the postpartum period is high, and these issues can negatively affect the quality of life for new mothers. After childbirth, sexual activity and interest in sex decrease. In a study conducted in Poland in 2017–2019, involving 398 women who completed the Female Sexual Functioning Index (FSFI) twice (before pregnancy and after childbirth), the results were striking. The study found that pregnancy and childbirth significantly (*p* < 0.001) reduced women’s sexual activity, impacting all six domains of the FSFI scale. Notably, 8.54% of women during pregnancy and 41.96% of women after childbirth had a score ≤ 26 points, indicating sexual dysfunction [4]. Additionally, in a multicenter longitudinal study conducted by Zhang et al. [7] in China from 2017 to 2019, in which a total of 217 women participated in examining sexual dysfunction during various stages of pregnancy, as well as six months postpartum, it was found that 97% (*n* = 211) of the women reported reduced levels of sexual activity during pregnancy. In comparison, 59.9% (*n* = 130) experienced a decrease in sexual activity six months after giving birth. According to the findings of the FSFI, the prevalence of sexual dysfunction in the first trimester was 100% (*n* = 217); in the second trimester, it was 97.23% (*n* = 211); in the third trimester, it was 96.21% (*n* = 203); and 6 months postpartum, it was 64.06% (*n* = 139). The high levels of sexual dysfunction appeared to be due to negative emotions, experiences, or personal perceptions regarding sexual behavior during pregnancy.

However, many other reasons can cause this phenomenon. Pre-pregnancy background, for example, may influence sexual function during pregnancy and the postpartum period. Yildiz [8] noticed a significant linear correlation between sexual function during pregnancy and the postpartum period, as well as pre-pregnancy sexuality. The author concluded that pre-pregnancy sexuality plays an essential role in maintaining sexual well-being during pregnancy and the puerperium. Additionally, Bond et al. [9] reported that sexual dysfunction, distress, and painful intercourse are common during the preconception period; however, participants often did not discuss their sexual experiences when talking about their plans to conceive. Another factor that influences sexual function during puerperium is breastfeeding, which can lead to several physiological changes in a woman’s body due to low levels of estrogen, progesterone, and androgens, along with high levels of prolactin and oxytocin. These changes may result in decreased vaginal lubrication, reduced blood vessel congestion, vaginal dryness, thinning of the vaginal epithelium, increased nipple sensitivity, and milk leakage. As a result, these breastfeeding-related changes can contribute to reduced sexual desire, increased pain during intercourse (dyspareunia), and overall sexual dissatisfaction. Furthermore, with breastfeeding, estrogen levels decrease, ultimately affecting sexuality, although this is not always absolute [10,11]. The existing literature on the relationship between breastfeeding and sexual function during childbirth presents partly contradictory findings. While some studies indicate that breastfeeding may have a positive impact on postpartum sexuality, attributed to increased breast tenderness and elevated oxytocin levels, other research identifies breastfeeding as a significant risk factor for postpartum sexual dysfunction. In the context of hormonal suppression, breastfeeding may lead to issues such as reduced frequency of sexual intercourse, dyspareunia (painful intercourse), and problems with lubrication and arousal, as well as diminished sexual desire [12]. Lack of partner support is another significant factor that negatively affects sexual function during the postpartum period. Research has shown that the quality of partnership and spouse support statistically reduces sexual function in all domains of the FSFI [13]. Postpartum women also complain of urinary incontinence, dyspareunia due to inadequate arousal after childbirth, and a feeling of organ mismatch [2,4,5]. This highlights the importance of understanding these factors, as well as other uninvestigated factors that contribute to these disorders.

Even though postpartum sexual health is a common concern, it is not often discussed during prenatal or postpartum care and has received little attention from either clinicians or researchers [12]. Research indicates that only a few postpartum women receive sufficient information regarding the changes in sexual function that occur after childbirth. Despite this, approximately two-thirds of women have discussed with their doctor when it is safe to resume sex [14]. In a phenomenological study conducted in Catalonia, Spain, in 2017, researchers revealed that many women did not feel ready for sexual intercourse following childbirth. Several factors inhibited their willingness to engage in sexual activity, including the new dynamics at home, various socio-cultural influences, and a lack of support from the healthcare system regarding this issue. Most participants reported a reduced libido and a negative body image, often resuming sexual activity before they felt truly ready. Additionally, they experienced fatigue and felt overwhelmed by the demands of caring for a newborn. This study concluded that support from partners and practical assistance with daily responsibilities was essential for fostering a meaningful relationship during this transition [15].

Although there is not enough research on women’s sexual contact during the early period of motherhood, the data to date indicate that women in the postpartum period need sexual contact [16]. Still, the way the birth developed and the interventions that had to be made or its complications, breastfeeding, and the relationship quality with their partner determine the frequency and nature of this contact [17,18]. Regarding sexual health studies in Greece, to our knowledge, there are no large-scale prospective studies that have thoroughly investigated the issue of sexual dysfunction after childbirth and during the postpartum period, as well as the factors that are possibly related to sexual dysfunction. Thus, the aim of this study was to investigate associated factors with postpartum sexual dysfunction during the puerperium period, with the hope of sparking a change in this trend. We hypothesize that sexual functioning may be negatively affected by factors such as low family or spousal support, low family income, past emotional disorders, being divorced, having an unplanned cesarean delivery, and an inability to breastfeed. Conversely, we believe that sexual functioning may be positively associated with having a positive body image, breastfeeding, high family income, spouse and/or family support, the absence of smoking and drinking, higher levels of physical activity, meeting birth expectations, and delivering full-term or early-term infants who did not require hospitalization in the Neonatal Unit.

## 2. Materials and Methods

### 2.1. Participants

Women who expressed their desire to participate in the study voluntarily were screened for eligibility based on the following inclusion and exclusion criteria. Inclusion criteria included age over 18 years and the time since childbirth not exceeding 12 months. Women who had given birth under 18 years of age, participated in a similar study, and denied giving the necessary information to the researchers were excluded from the study.

### 2.2. Study Design

For the preparation of this study, the detailed research protocol, the relevant information, and the consent form for the participants were submitted to the Research Ethics Committee of the Department of Medicine of the Aristotle University of Thessaloniki (AUTH), Greece. The study received approval on 14 February 2023 (No. Prot.: 89/2023). Subsequently, an invitation to participate in the study was announced to postpartum women and new mothers through websites and forums on motherhood. In this proposal, the objectives, methodological approach, confidentiality, and anonymity were described in detail [By the General Data Protection Regulation “GDPR”, European Union Regulation (EU) 2016/679]. Women who voluntarily wanted to participate were asked to fill in two questionnaires: a general questionnaire regarding demographic and other personal information about the postpartum period and the Female Sexual Functioning Index (FSFI).

### 2.3. General Questions Regarding Demographic and Other Personal Information

Participants were asked to complete a questionnaire that gathered demographic and other relevant information, including gender identity, age, sexual orientation, smoking habits, alcohol consumption, body image, educational level, annual family income, employment status, and marital status. They were also asked about the support they receive from their spouse or partner, as well as their family environment. Additionally, participants provided details regarding childbirth, including the type, timing, and other characteristics related to the experience.

### 2.4. Female Sexual Functioning Index

The FSFI was designed to be a user-friendly and reliable tool for assessing the multidimensional nature of female sexual function. The FSFI assesses a total of 6 domains of female sexual function: (1) desire, (2) arousal, (3) lubrication, (4) orgasm, (5) satisfaction, and (6) pain. The FSFI questionnaire consists of 19 multiple choice questions, and the responses are based on a 5-point Likert-type cumulative rating scale. Each of the 19 questions has a specific multiplying factor ranging from 0.3 to 0.6 and corresponds to one of the six domains above. In this way, each individual domain has a maximum score of 6, while the total maximum FSFI score is 36 points. In contrast, the minimum score for each domain ranges from 0 to 1.2. Thus, the overall minimum score for the FSFI is 2 points [19].

Furthermore, research has shown that, when the total FSFI score is 26 points or lower, it more accurately reflects the presence of sexual dysfunction (specificity = 0.733; sensitivity = 0.889). However, it is also recommended to consider the individual scores for each domain, both within the model and conceptually, to enhance the accuracy of differential diagnoses among the various types of female sexual dysfunction [20]. In this study, the Greek version of the FSFI was used. Five native speakers of Greek, including a psychologist, a urologist, a gynecologist, and two translators, translated and weighed the English version of the FSFI into Greek, ensuring its accuracy and reliability [21]. The different translations were then compared and merged into a single translated version of the FSFI and subsequently translated back (from Greek to English) by an English native speaker of Greek. This second English version of the questionnaire was compared with the original English version. Then, the new Greek version was tested on five individuals to check comprehension, interpretation, and cultural relevance. After examining the results for cognitive comprehension, the final Greek version was ready for statistical validation.

To weigh and validate the questionnaire, the translated version of the FSFI was administered to 99 healthy women and 18 women with sexual dysfunction. The results showed that the Greek version of the FSFI had a high Cronbach’s alpha reliability index (0.92) [21], while the internal consistency was comparable to the corresponding studies by Rosen et al. [19] and Rillon et al. [22], in which the Cronbach’s alpha was 0.88 and 0.95, respectively, with a range of 0.89–0.97 [19,22].

### 2.5. Statistical Analysis

The IBM Statistical Package for Social Sciences (IBM Corp. Released 2022. IBM SPSS Statistics for Windows, Version 28.0., Armonk, NY, USA: IBM Corp) was used for statistical analysis of the results. The analysis of the results was initially based on descriptive statistics to extract the frequencies (v) and percentages (%) of the variables used as quantitative risk factors. Each variable had a range of two to six possible answers, and thus, two to five groups of postpartum women emerged. Therefore, frequencies and percentages were utilized to compare the results among these groups. For the analysis of the FSFI subscales, the data were presented as the mean (M) ± standard deviation (SD), along with the minimum (Min) and maximum (Max) values for each subscale. The M and SD were also used in tables that examined the effects of risk factor variables on sexual function. The relationship of various socio-demographic and other factors with the level of sexual functioning of postpartum women was examined with the *t*-test and the one-way ANOVA test. A t-test was used for comparisons involving two groups, such as the variable “first child”, which had responses of “Yes” or “No”. For comparisons involving three or more groups, such as the variable “age group”, which had four possible responses, ANOVA was utilized. The data were analyzed using analysis of variance with Bonferroni post hoc calculation. The significance level for accepting or not the existence of a statistically significant difference obtained for all statistical tests was set at *p* < 0.05.

## 3. Results

### 3.1. Participants’ Characteristics

A total of 336 heterosexual postpartum women voluntarily consented to participate in the current study. The patients’ demographic and other personal characteristics are shown in Table 1, Table 2, Table 3 and Table 4.

### 3.2. FSFI Scale Results

The descriptive results for the six subscales and the total score of the FSFI scale are given in Table 5. The results show that the women during the postpartum period had moderate sexual functioning (M = 20.8, SD = 11.3). Of the dimensions of sexual functioning, the lowest scores appeared in the dimensions of arousal (M = 3.1, SD = 2.2) and orgasm (M = 3.2, SD = 2.4), while the highest scores appeared in the dimensions of satisfaction (M = 3.9, SD = 1.9) and pain (M = 3.6, SD = 2.4).

### 3.3. Risk Factors for Sexual Dysfunction in Postpartum Women

Table 6 presents the findings of the analysis regarding the difference in sexual functioning of postpartum women in terms of their demographic and other data. These findings showed that the level of sexual functioning of the postpartum women differed significantly compared to their age group (F = 2.656, *p* = 0.033), compared to their educational level (F = 2.806, *p* = 0.026), and compared to annual family income (F = 2.726, *p* = 0.020). The findings showed that women over 40 years of age (M = 13.3, SD = 11.5) had a significantly lower level of sexual functioning compared to younger women. Similarly, women with a Master’s degree (M = 17.9, SD = 11.9) had a significantly lower level of sexual functioning compared to women who had not finished school (M = 26.4, SD = 9.7) and high school graduates (M = 23.9, SD = 10.4). Moreover, women with an annual family income less than 40.000 EUR (M = 12.1, SD = 9.5) had a lower level of sexual functioning compared to other women. While women with a positive body image (M = 26.0, SD = 10.5) showed significantly higher levels of sexual functioning than those with a lower body image (Table 6).

In addition, the findings revealed significant differences in the level of sexual functioning among women based on the support they received from their partners and family environments. Specifically, the analysis showed that sexual functioning was correlated with partner support (F = 4.225, *p* = 0.006) and family support (F = 2.963, *p* = 0.032). Women with abusive partners or those who lacked support (M = 10.2, SD = 6.4) exhibited significantly lower levels of sexual functioning compared to women who had supportive partners, even if the support was limited (M = 19.7, SD = 11.2). Furthermore, women with completely supportive partners reported an even higher level of sexual functioning (M = 21.9, SD = 11.4). In terms of family support, women who came from unsupportive family environments (M = 16.2, SD = 11.3) also demonstrated significantly lower levels of sexual functioning compared to those with completely supportive family environments (M = 22.2, SD = 11.3) (Table 7).

In addition, Table 8 presents the findings of an analysis regarding differences in sexual functioning among postpartum women based on various childbirth-related characteristics. The results indicate that the level of sexual functioning varies significantly depending on the amount of time since childbirth (F = 6.337, *p* = 0.000) and the women’s expectations from the childbirth experience (t = −1.991, *p* = 0.047).

Specifically, women who gave birth less than one month before the survey (M = 13.1, SD = 11.7) reported a significantly lower level of sexual functioning compared to those who gave birth 4–6 months prior (M = 22.8, SD = 11.2) and those who gave birth 6–12 months prior (M = 22.4, SD = 10.8). Furthermore, women who felt their expectations from childbirth were met (M = 21.6, SD = 11.5) reported a significantly higher level of sexual functioning than those who experienced a traumatic childbirth (M = 18.8, SD = 10.8).

Moreover, the results showed a significant difference in sexual functioning based on breastfeeding status, especially for women who had cesarean sections (F = 6.570, *p* = 0.000). Women who desired to breastfeed but were unable to produce milk (M = 18.9, SD = 11.8) and those who were still breastfeeding (M = 18.3, SD = 11.8) had significantly lower sexual functioning compared to women who chose not to breastfeed (M = 26.0, SD = 8.5) and those who breastfed for a short period (M = 22.8, SD = 10.6) (Table 8).

Finally, Table 9 shows that the level of sexual functioning of women after childbirth does not differ to a statistically significant degree compared to the women’s health status (t = 1.571, *p* = 0.117) and the individual (t = 0.299, *p* = 0.765) and family (t = −0.492, *p* = 0.629) histories of emotional disorders.

## 4. Discussion

The aim of this study was to investigate the associated factors with postpartum sexual dysfunction during the puerperium period, with the hope of sparking a change in this trend. We hypothesized that factors such as body image, breastfeeding, spouse and/or family support, family income, smoking, drinking, levels of physical activity, birth expectations, delivering full-term or early-term infants who did or did not require hospitalization in the Neonatal Unit, and others will have a significant impact on the sexual function of postpartum women.

Our study showed that women scored an average of 20.8 points on the FSFI, and thus, their level of sexual functioning was characterized as moderate. Interestingly, of the dimensions of sexual functioning, the lowest scores appeared in the dimensions of arousal (M = 3.1, SD = 2.2) and orgasm (M = 3.2, SD = 2.4), while the highest scores appeared in the dimensions of satisfaction (M = 3.9, SD = 1.9) and pain (M = 3.6, SD = 2.4). These results underscore that postpartum women may feel satisfaction during sexual activity, but they experience vaginal pain, have difficulty becoming aroused, and struggle to reach orgasm. A previous prospective study found that the FSFI scores deteriorated by the third trimester and did not return to the levels observed in early pregnancy, even six months after childbirth. This suggests the presence of sexual dysfunction among postpartum women (*p* = 0.017). Additionally, the type of delivery had a significant effect on the total FSFI scores but not a statistical one. Women who had a cesarean section reported higher FSFI scores than those who had a vaginal delivery, with mean scores of 27.7 for the cesarean group compared to 23.7 for the vaginal delivery group (*p* = 0.127). This indicates that women who underwent cesarean sections may experience better sexual function [23]. Furthermore, their study showed that, although body image did not change significantly during pregnancy, it worsened during the postpartum period (*p* = 0.01), significantly affecting women’s sexual functioning. In agreement, Zhang et al. [7] found that a woman’s body image, as well as factors such as sleep quality, fatigue, pain, age, income, previous pregnancies, type of delivery, breastfeeding, postpartum depression, and social support, did not significantly impact sexual function at 6 months postpartum. Additionally, Matthies et al. [13] observed that the quality of partnership, breastfeeding, high maternal education, and maternal depressive symptoms were significantly associated with postpartum FSFI scores. Similarly, our study showed that there were no significant differences in sexual functioning based on the type of delivery (*p* = 0.783). In contrast, women with a positive body image and spouse support showed significantly higher levels of sexual functioning than those with a lower body image (*p* = 0.014) and those with abusive partners or those who lacked support (*p* = 0.006). Meanwhile, women who came from unsupportive family environments also demonstrated significantly lower levels of sexual functioning compared to those with completely supportive family environments (*p* = 0.032).

Our study also showed that higher age (*p* = 0.033), lower educational level (*p* = 0.026), lower annual income (*p* = 0.020), breastfeeding (*p* = 0.000), and traumatic childbirth experience (*p* = 0.047) appeared to influence the scores on the FSFI scale. Dahlgren et al. [6] identified several statistically significant risk factors for sexual inactivity one year after delivery, including an age equal to or higher than 35 years, experiencing at least second-degree perineal injuries, the duration of breastfeeding, and having poor physical health during pregnancy. Matthies et al. [13] found that women who stopped breastfeeding or never breastfed had the highest FSFI scores. Thus, women who exclusively breastfeed and those with poor partner communication are more likely to experience sexual dysfunction in the first four months postpartum. Furthermore, Avery et al. [24] found a slight negative association between the physiological aspects of breastfeeding and sexuality. However, most women reported that breastfeeding did not significantly impact their sexual relationships with their partners. Many women indicated that their partners had a slightly positive perception of the relationship between breastfeeding and sexuality. Additionally, breastfeeding mothers generally expressed little concern that their sexual activity would harm their milk supply or their ability to breastfeed. These findings show partial agreement with our results. Women over the age of 40 who wanted to breastfeed but were unable to produce milk or who continued breastfeeding had significantly lower sexual functioning compared to those who chose not to breastfeed or those who only breastfed for a short period. In contrast, our study did not reveal any significant impact of physical activity levels on the FSFI or investigate the experience of perineal injuries in several domains of sexual function.

Moreover, in our study, it was found that sexual functionality levels vary significantly depending on the duration of childbirth but not on the fact that women expected their first child (*p* = 0.074). Typically, women who had given birth just one month prior exhibited lower levels of sexual functionality compared to those who had given birth more recently (*p* = 0.000). During the postpartum period, many women do not experience or feel a strong sexual desire. Engaging in sexual intercourse after childbirth can raise fears and concerns about potential pain or the possibility of becoming pregnant again. Therefore, the amount of time that has passed since childbirth can influence a woman’s level of sexual desire and functionality. Mekonnen et al. [25] showed that an early initiation of sexual intercourse after childbirth was 6.12 times more likely in women living in urban areas than in rural areas. Women in equal relationships with their partners were about 2.26 times more likely to resume sexual intercourse within six weeks after childbirth. The desire for sexual intercourse in men was about 2.66 times higher than that in women. Also, having a child as part of family planning led to approximately 2.72 times higher chances of women resuming early sexual intercourse compared to women for which pregnancy was a random event [25]. Likewise, Edosa Dirirsa et al. [26] showed that only 31.6% of postpartum women returned to their early sexual activity 6 weeks after delivery [26].

Lastly, our study has several strengths and limitations. Firstly, our study is one of the first to evaluate sexual function in postpartum women during the first year after delivery. Secondly, we identified several significant risk factors that impact postpartum sexual function, as measured by the FSFI. These factors include age, breastfeeding, body image, the time elapsed since labor, and support from the spouse or family. The absence of risk factors of variables such as dyspareunia and vaginal lacerations, which may have a significant impact on sexual function and the FSFI scores before and during pregnancy to compare the FSFI scores in three different periods (pre-pregnancy, during pregnancy, and postpartum) may be considered limitations of our study. Future studies should focus on training postpartum healthcare staff to provide appropriate sexual guidance after childbirth. Specifically, it is essential to investigate the current practices that offer this guidance. Additionally, the healthcare staff involved in issues related to sexual function and the puerperium should participate in more comprehensive educational programs to enhance their qualifications. Given that both women and healthcare providers often hesitate to discuss sexual concerns during pregnancy and the postpartum period, possibly due to women feeling uncomfortable bringing up these topics and lack of proper training to effectively obtain a sexual history and provide appropriate guidance or referrals to specialists in sexology from healthcare providers, these studies are of great significance [12,27]. It is essential that mothers, especially new mothers who are faced with the new reality of motherhood, feel comfortable discussing issues related to sexual activity during pregnancy and after childbirth. In this way, emerging risk factors can be easily identified, for example, by using appropriate questionnaires that directly address female sexuality or by conducting appropriately structured interviews and resolved to the greatest extent possible.

## 5. Conclusions

In summary, this study is one of the first to evaluate sexual function in women during the first year postpartum in Greece. The results indicated that many postpartum women in Greece experience sexual dysfunction during this period. The study highlighted several uncertain or little-investigated risk factors that contribute to this issue, including breastfeeding, body image, having a first child, and the duration of labor. Larger longitudinal studies are necessary to investigate the long-term effects of pregnancy and childbirth on women’s sexual function after giving birth [12,27].

## Figures and Tables

**Table 1:** Demographic, lifestyle, and body image data of postpartum women (N = 336).

		v (%)
Age group (years)	(a) 18–25	33 (9.8%)
	(b) 26–30	96 (28.6%)
	(c) 31–35	124 (36.9%)
	(d) 36–40	65 (19.3%)
	(e) Over 40	18 (5.4%)
Educational level	(a) No education/unfinished school	7 (2.1%)
	(b) High school diploma	70 (20.8%)
	(c) Vocational Training Institute diploma	79 (23.5%)
	(d) Bachelor’s degree	133 (39.6%)
	(e) Master’s degree	47 (14.0%
Annual family income	(a) Up to 5000 EUR	52 (15.5%)
	(b) 5001–10,000 EUR	76 (22.6%)
	(c) 10,001–20,000 EUR	129 (38.4%)
	(d) 20,001–30,000 EUR	46 (13.7%)
	(e) 30,001–40,000	16 (4.8%)
	(f) 40,001 and over	17 (5.1%)
Employment status	(a) Not working	141 (42.0%)
	(b) Part-time working	52 (15.5%)
	(c) Full-time working	143 (42.6%)
Type of employment	(a) Housewife	123 (36.6%)
	(b) Self-employed	43 (12.8%)
	(c) Private employ	137 (40.8%)
	(d) Public employ	33 (9.8%)
Marital status	(a) Single	3 (0.9%)
	(b) In a relationship/Engaged	14 (4.2%)
	(c) Married	313 (93.2%)
	(d) Divorced	6 (1.8%)
Smoking	(a) No, I never smoked	151 (44.9%)
	(b) No, I cut it because of the child	67 (19.9%)
	(c) Yes, occasionally	46 (13.7%)
	(d) Yes, systematically	72 (21.4%)
Drinking	(a) Rarely, less than 1 time per week	298 (88.7%)
	(b) A little, 1–2 times per week	30 (8.9%)
	(c) Moderately, 3–4 times per week	4 (1.2%)
	(d) Quite often, almost daily	4 (1.2%)
Physical Activity	(a) No, I don’t have time	201 (59.8%)
	(b) No, I don’t like sports	62 (18.5%)
	(c) Yes, when I have time	61 (18.2%)
	(d) Yes, systematically	12 (3.6%)
Body image	(a) Negatively, I look at myself and I get disappointed.	79 (23.5%)
	(b) Moderately negative	86 (25.6%)
	(c) Moderately positive	136 (40.5%)
	(d) Positively, I really like myself!	35 (10.4%)

Note: Data are expressed as frequencies (v) and percentages (%).

**Table 2:** Data on support from the spouse/partner and support from the family environment.

		v (%)
The other half, after birth, is for the woman.	(a) Abusive	1 (0.3%)
	(b) Not at all supportive	5 (1.5%)
	(c) Slightly supportive	15 (4.5%)
	(d) Less supportive than she would like	87 (25.9%)
	(e) Completely supportive	228 (67.9%)
The family environment, after birth, is for the woman	(a) Not at all supportive	9 (2.7%)
	(b) Slightly supportive	29 (8.6%)
	(c) Less supportive than she would like	85 (25.3%)
	(d) Completely supportive	213 (63.4%)

Note: Data are expressed as frequencies (v) and percentages (%).

**Table 3:** Data about the childbirth.

		v (%)
Time of labor before the study enrollment	(a) Less than a month	25 (7.4%)
	(b) 1–2 months	22 (6.5%)
	(c) Over 2 and less than 4 months	54 (16.1%)
	(d) Over 4 and less than 6 months	60 (17.9%)
	(e) Over 6 to 12 months	175 (52.1%)
First child	(a) No	139 (41.4%)
	(b) Yes	197 (58.6%)
Type of delivery	(a) Planned C-section	101 (30.1%)
	(b) Emergency C-section	58 (17.3%)
	(c) Normal	121 (36.0%)
	(d) Vacuum Extraction Delivery	13 (3.9%)
	(e) She tried naturally but had a caesarean section	43 (12.8%)
The baby was born	(a) Early term, required prolonged hospitalization in the Neonatal Unit	10 (3.0%)
	(b) Early term but did not require hospitalization in the Neonatal Unit	34 (10.1%)
	(c) full term, but required hospitalization in the Neonatal Unit	13 (3.9%)
	(d) Full term	265 (78.9%)
	(e) Early term, required brief hospitalization in Neonatal Unit	14 (4.2%)
Did the birth experience meet your expectations about it?	(a) No, the way I gave birth was a traumatic experience for me.	95 (28.3%)
	(b) Yes, it was a wonderful experience.	241 (71.7%)
Breastfeeding	(a) I didn’t breastfeed by choice	33 (9.8%)
	(b) I wanted to breastfeed, but I didn’t have milk	30 (8.9%)
	(c) I breastfed for a while	126 (37.5%)
	(d) I’m still breastfeeding	147 (43.8%)

Note: Data are expressed as frequencies (v) and percentages (%).

**Table 4:** Health status and emotional disorder history.

		v (%)
Do you have a medical condition that requires treatment?	(a) No	268 (79.8%)
	(b) Yes	68 (20.2%)
Have you had any emotional disorders in the past? (e.g., depression, anxiety disorder, etc.)	(a) No	234 (69.6%)
	(b) Yes, but I didn’t need treatment.	72 (21.4%)
	(c) Yes, and I received treatment.	30 (8.9%)
Is there a family history of any emotional disorders, such as depression, anxiety disorders, or bipolar disorder?	(a) No	228 (67.9%)
	(b) Yes	108 (32.1%)

Note: Data are expressed as frequencies (v) and percentages (%).

**Table 5:** Results of the descriptive analysis for the sexual functioning assessment subscales and the overall level of sexual functioning of postpartum women.

	M	SD	Min	Max
Desire	3.4	1.5	1.2	6.0
Arousal	3.1	2.2	0.0	6.0
Lubrication	3.5	2.4	0.0	6.0
Orgasm	3.2	2.4	0.0	6.0
Satisfaction	3.9	1.9	0.8	6.0
Pain	3.6	2.4	0.0	6.0
Sexual function	20.8	11.3	2.0	36.0

Note: Μ: mean; SD: standard deviation; Min: minimum value; Max: maximum value.

**Table 6:** Demographic, lifestyle, and body image data of postpartum women and sexual function.

		Sexual Function			Test	*p*-Value
		M	SD	CI (Lower Bound/ Upper Bound)		
Age group (years)	(a) 18–25	23.6	9.9	20.09/27.11	2.656	0.033
	(b) 26–30	21.6	11.2	19.33/23.86		
	(c) 31–35	20.6	11.7	18.52/22.68		
	(d) 36–40	20.5	10.9	17.79/23.20		
	(e) Over 40	13.3	11.5	7.58/19.01		
Educational level	(a) No education/unfinished school	26.4	9.7	17.42/35.37	2.806	0.026
	(b) High school diploma	23.9	10.4	21.42/26.38		
	(c) Vocational Training Institute diploma	21.0	11.7	18.38/23.62		
	(d) Bachelor’s degree	19.8	11.2	17.87/21.72		
	(e) Master’s degree	17.9	11.9	14.40/21.39		
Annual family income	(a) Up to 5000 EUR	20.6	11.1	17.51/23.69	2.726	0.020
	(b) 5001–10,000 EUR	20.9	11.1	18.36/23.43		
	(c) 10,001–20,000 EUR	21.7	11.4	19.71/23.68		
	(d) 20,001–30,000 EUR	22.4	11.3	19.04/25.75		
	(e) 30,001–40,000	17.5	12.0	11.10/23.89		
	(f) 40,001 and over	12.1	9.5	7.21/16.98		
Employment status	(a) Not working	20.4	11.1	18.55/22.24	0.425	0.654
	(b) Part-time working	22.1	11.7	18.84/25.35		
	(c) Full-time working	20.8	11.5	18.89/22.70		
Type of employment	(a) Housewife	19.9	11.0	17.93/21.86	1.693	0.168
	(b) Self-employed	20.6	11.5	17.06/24.13		
	(c) Private employ	22.3	11.2	20.40/24.19		
	(d) Public employ	18.1	12.5	13.66/22.53		
Marital status	(a) Single	7.9	8.2	12.47/28.27	1.338	0.262
	(b) In a relationship/Engaged	21.5	11.5	14.86/28.14		
	(c) Married	20.9	11.3	19.64/22.15		
	(d) Divorced	21.7	11.9	9.21/34.18		
Smoking	(a) No, I never smoked	19.6	11.2	17.79/21.40	2.001	0.114
	(b) No, I cut it because of the child	20.1	11.9	17.19/23.00		
	(c) Yes, occasionally	22.1	12.0	18.53/25.66		
	(d) Yes, systematically	23.2	10.4	20.75/25.64		
Drinking	(a) Rarely, less than 1 time per week	20.6	11.4	19.30/21.90	1.755	0.156
	(b) A little, 1–2 times per week	20.1	11.5	15.80/24.39		
	(c) Moderately, 3–4 times per week	31.2	5.3	22.76/39.63		
	(d) Quite often, almost daily	28.2	7.9	15.62/40.77		
Physical Activity	(a) No, I don’t have time	20.6	11.0	19.07/22.13	2.554	0.054
	(b) No, I don’t like sports	17.9	12.0	14.85/20.94		
	(c) Yes, when I have time	23.5	11.5	20.55/26.44		
	(d) Yes, systematically	26.3	8.3	21.02/31.57		
Body image	(a) Negatively, I look at myself and I get disappointed.	20.0	10.6	17.63/22.37	3.615	0.014
	(b) Moderately negative	18.8	11.9	16.24/21.35		
	(c) Moderately positive	21.2	11.3	19.28/23.11		
	(d) Positively, I really like myself!	26.0	10.5	22.39/29.61		

Note: The relationships of various socio-demographic and other factors with the level of sexual functioning of postpartum women were examined with the *t*-test and the one-way ANOVA test. A *t*-test was used for comparisons involving two groups. For comparisons involving three or more groups, ANOVA was utilized. Μ: mean; SD: standard deviation; CI: 95% confidence interval; significant at *p* < 0.05.

**Table 7:** Data on support from the spouse/partner and support from the family environment and sexual function.

		Sexual Function			Test	*p*-Value
		M	SD	CI (Lower Bound/ Upper Bound)		
The other half, after birth, is for the woman.	Abusive/Not at all supportive	10.2	6.4	3.48/16.91	4.225	0.006
	Slightly supportive	14.8	9.2	13.49/25.90		
	Less supportive than she would like	19.7	11.2	17.31/22.08		
	Completely supportive	21.9	11.4	20.41/23.38		
The family environment, after birth, is for the woman	Not at all supportive	16.2	11.3	7.51/24.88	2.963	0.032
	Slightly supportive	19.1	10.7	15.03/23.17		
	Less supportive than she would like	18.5	11.3	14.20/22.79		
	Completely supportive	22.2	11.3	20.67/23.72		

Note: The relationships of the factors with the level of sexual functioning of postpartum women were examined with the *t*-test and the one-way ANOVA test. A *t*-test was used for comparisons involving two groups. For comparisons involving three or more groups, ANOVA was utilized. Μ: mean; SD: standard deviation; CI: 95% confidence interval; significant at *p* < 0.05.

**Table 8:** Data about childbirth and sexual function.

		Sexual Function			Test	*p*-Value
		M	SD	CI (Lower Bound/ Upper Bound)		
Time of labor during the study enrollment	(a) Less than a month	13.1	11.7	8.27/17.93	6.337	0.000
	(b) 1–2 months	14.8	12.8	9.12/20.47		
	(c) Over 2 and less than 4 months	19.4	10.3	16.58/22.20		
	(d) Over 4 and less than 6 months	22.8	11.2	19.90/25.69		
	(e) Over 6 to 12 months	22.4	10.8	20.78/24.01		
First child	(a) No	22.1	11.4	20.18/24.01	1.795	0.074
	(b) Yes	19.9	11.2	18.32/21.47		
Type of delivery	(a) Planned C-section	21.7	11.8	19.37/24.02	0.435	0.783
	(b) Emergency C-section	20.8	10.7	17.98/23.61		
	(c) Normal	20.8	11.5	18.73/22.87		
	(d) Vacuum Extraction Delivery	18.7	11.6	11.69/25.71		
	(e) She tried naturally but had a caesarean section	19.4	11.0	16.01/22.78		
The baby was born	(a) Early term, required prolonged hospitalization in the Neonatal Unit	20.3	8.0	14.57/26.02	0.434	0.784
	(b) Early term but did not require hospitalization in the Neonatal Unit	20.3	12.3	16.00/24.59		
	(c) full term, but required hospitalization in the Neonatal Unit	18.9	10.1	12.79/25.00		
	(d) Full term	21.1	11.5	19.70/22.49		
	(e) Early term, required brief hospitalization in Neonatal Unit	17.7	9.5	12.21/23.18		
Did the birth experience meet your expectations about it?	(a) No, the way I gave birth was a traumatic experience for me.	18.8	10.8	16.60/21.00	−1.991	0.047
	(b) Yes, it was a wonderful experience.	21.6	11.5	20.14/23.05		
Breastfeeding	(a) I didn’t breastfeed by choice	26.0	8.5	22.99/29.01	6.570	0.000
	(b) I wanted to breastfeed, but I didn’t have milk	18.9	11.8	14.49/23.30		
	(c) I breastfed for a while	22.8	10.6	20.93/24.66		
	(d) I’m still breastfeeding	18.3	11.8	16.37/20.22		

Note: The relationships of the factors with the level of sexual functioning of postpartum women were examined with the *t*-test and the one-way ANOVA test. A *t*-test was used for comparisons involving two groups. For comparisons involving three or more groups, ANOVA was utilized. Μ: mean; SD: standard deviation; CI: 95% confidence interval; significant at *p* < 0.05.

**Table 9:** Data about health status, emotional disorder history, and sexual function.

		Sexual Function			Test	*p*-Value
		M	SD	CI (Lower Bound/ Upper Bound)		
Treatment for a disease	(a) No	21.3	11.3	19.94/22.65	1.571	0.117
	(b) Yes	18.9	11.5	16.11/21.68		
Personal history of emotional disorder in the past	(a) No	21.1	11.5	19.61/22.58	0.299	0.765
	(b) Yes, but I didn’t need treatment.	20.3	11.3	17.64/22.95		
	(c) Yes, and I received treatment.	19.3	10.5	15.37/23.22		
Family history of emotional disorder in the past	(a) No	20.9	11.4	19.41/22.38	−0.492	0.629
	(b) Yes	20.5	11.3	18.34/22.65		

Note: The relationships of the factors with the level of sexual functioning of postpartum women were examined with the *t*-test and the one-way ANOVA test. A *t*-test was used for comparisons involving two groups. For comparisons involving three or more groups, ANOVA was utilized. Μ: mean; SD: standard deviation; CI: 95% confidence interval; significant at *p* < 0.05.

## Data Availability

The data presented in this study are available upon request from the corresponding author. The data are not publicly available due to ethical restrictions.

## Public Involvement Statement

No public involvement in any aspect of this research.

## Guidelines and Standards Statement

This manuscript was drafted against the STROBE for cross-sectional study research.
